# Linking Obesity and Depression Through the Gut–Brain Axis: The Impact of Short-Chain Fatty Acids and Inflammation

**DOI:** 10.3390/nu18060898

**Published:** 2026-03-12

**Authors:** Vlad Ionuț Vlăsceanu, Sergiu Timofeiov, Alin Constantin Pînzariu, Radu Petru Soroceanu, Madalina Maxim, Lucian Ambrosie, Ancuța Andreea Miler, Tudor Cojocaru, Giulia Mihaela Cojocaru, Sebastian Marian Leonte, Alexandra Gabriela Trofin, Daniel Vasile Timofte

**Affiliations:** 1 Grigore T. Popa University of Medicine and Pharmacy Iasi, 700115 Iasi, Romania; vlasceanu.vlad@yahoo.com (V.I.V.); tudose.timofeiov@umfiasi.ro (S.T.); madalynamaxim@yahoo.com (M.M.); lucian.ambrosie@hse.ie (L.A.); ancuta-andreea_a_miler@d.umfiasi.ro (A.A.M.); tudorcoj@yahoo.com (T.C.); pinzariu_giulia@yahoo.com (G.M.C.); mg-rom-33565@students.umfiasi.ro (S.M.L.); mg-rom-31141@students.umfiasi.ro (A.G.T.); daniel.timofte@umfiasi.ro (D.V.T.); 2 Department of Surgery, Sf. Spiridon County Emergency Clinical Hospital, 700111 Iasi, Romania

**Keywords:** obesity, SCFAs, metal health, microbiota, depression, anxiety, metabolic surgery

## Abstract

Obesity is a major public health problem that puts pressure on healthcare systems globally. The purpose of this narrative review is to summarize and analyse recent research on the bidirectional link between obesity and mental health, focusing on the biological, behavioural, dietary, emotional, and metabolic mechanisms arising from gut microbiota interactions. Epidemiological association between obesity and mental health disorders, especially depression and anxiety, often occurs bidirectionally, reinforcing each other. Low-grade systemic inflammation is a condition typically found in obesity, being a fundamental element of neuropsychiatric disorders. Considered the main energy substrate for colon cells, SCFAs are synthesized in the intestine and exert important local effects by reducing both local and systemic inflammation. The intestinal microbiota maintains this homeostasis through the SCFAs it produces. The combined impact of the increased intestinal permeability, immune activation, and disrupted metabolism of SCFAs and tryptophan contributes to the onset and progression of depression and anxiety, as well as to significant cognitive dysfunction, especially in obese individuals. Understanding the mechanisms by which microbiota metabolites influence brain development, neuroplasticity, and behaviour could pave the way for new and innovative therapeutic strategies for the treatment of obesity and depression. Conclusions: The association of these pathologies is not coincidental, as they coexist through overlapping biological pathways that they partially or completely share. The main pathway involved is formed by the brain–gut axis and its mediators (SCFAs).

## 1. Introduction

Obesity is a major public health problem that puts pressure on healthcare systems globally. It affects over 650 million adults worldwide, and prevalence rates are increasing in both developed and developing countries [1,2]. Beyond the cardiometabolic consequences that have been well documented over the past decades, obesity is strongly associated with mental health disorders, especially depression and anxiety [3,4,5]. The relationship between obesity and mental health is a bidirectional one, based on the fact that obesity increases the risk of depression by approximately 30–70%. Depressive symptoms may be seen as predictors of weight gain in the future [4,6].

Several mechanisms have been proposed to explain the complex interaction between obesity and mental health. Mild chronic systemic inflammation, hypothalamic–pituitary–adrenal (HPA) axis dysfunction, gut microbiota dysbiosis, and brain reward circuit dysfunction are the main pathological processes that might be implicated in the interaction of obesity with mental health [7,8,9,10]. A number of disorders, such as emotional eating and bulimia-like disorders, may serve as behavioural elements that influence and perpetuate the relationship between weight gain and emotional disorders [11,12].

The purpose of this narrative review is to summarize and analyse recent literature on the bidirectional link between obesity and mental health, focusing on the biological, behavioural, dietary, emotional, and metabolic mechanisms driven by gut microbiota interactions. This article emphasizes the negative roles of gut dysbiosis and intestinal permeability in modulating metabolic and affective disorders. Through this integrated approach, we aim to present the possible mechanisms that could link obesity to mental health, but also to highlight possible clinical implications of this connection.

## 2. Materials and Methods

This narrative review was conducted to explore the recent findings on the bidirectional relationship between obesity and mental health in patients diagnosed with both comorbidities through a synthesis of recent literature. We considered it important to include studies that provided a deep understanding of the link between the microbiome and metabolic disorders, the importance of personalized modulation of the gut microbiome, and those that addressed important scientific gaps. We adopted a narrative synthesis approach, in accordance with established methodological guidelines for non-systematic reviews in complex and emerging fields, grounded in the principles of the SANRA scale (Scale for the Quality Assessment of Narrative Review Articles). The objective of our work is to draw attention to the psychosocial and socio-economic implications that the physician must consider when choosing a pharmacological or surgical approach to obesity management, as well as the emerging risks of procedures such as bariatric surgery in the development and onset of severe postoperative psychiatric disorders.

This integrative approach, supported by recent scientific literature, enabled us to justify the importance of a dynamic perspective on the impact of obesity and bariatric surgery on SCFA production and to identify a literature search strategy that would present relevant and innovative aspects.

This paper summarizes the current state of knowledge on the interactions between SCFAs, obesity, and mental health, adopting an association-based language to faithfully reflect the heterogeneity of human data and the critical need to develop future longitudinal and standardized clinical protocols. In the absence of extensive human evidence, our review is largely based on preclinical data from animal models and other narrative or systematic reviews.

### 2.1. Search Strategy

We performed a comprehensive bibliographic search to identify relevant articles regarding the role of SCFAs in the occurrence of mental disorders, with a particular focus on depressive disorder, anxiety, and mood disorders. As this work is a narrative review, no strict inclusion criteria were established for selecting articles. Searches were conducted in electronic databases such as PubMed, MEDLINE, and Google Scholar, using keywords, and the results were focused on studies published between 2020 and 2025. Focusing on the literature published in recent years enabled us to select data from a multitude of studies based on the criteria of extended clinical applicability and new discoveries in the gut–brain axis. In addition, recent studies provide a comprehensive overview of the various factors that could disrupt the synthesis of SCFAs and influence pre-existing mental disorders. Based on titles and abstracts, we excluded conference abstracts, editorials, and studies not written in English. We also reviewed the reference lists of scientific articles included in the narrative review to align them with the interest of justifying the formulated hypothesis.

Search terms included combinations of the following keywords: “obesity,” “depression,” “anxiety,” “bariatric surgery,” “gut microbiota,” “gut–brain axis,” “gut permeability,” “leaky gut,” “low-grade inflammation,” and “emotional eating.” We applied Boolean operators (AND, OR, NOT) to refine searches (e.g., “obesity AND depression AND (gut microbiota OR leaky gut syndrome)”) and delineate the intersection of psychiatric and gastroenterological pathology, thereby enabling meaningful correlations for the quality of the narrative analysis. Grey literature (e.g., WHO report) was included as it was considered relevant for medical discussions and for justifying the epidemiological context.

### 2.2. Inclusion Criteria

Studies considered eligible were included in the narrative analysis if they were published in peer-reviewed journals between January 2020 and December 2025, written in English, and focused on adult human populations. Significant viewpoints consistent with our research were included, leading to the referencing of articles prior to 2020 when deemed relevant. We considered it important to include studies that provided a deep understanding of the link between the microbiome and metabolic disorders, the importance of personalized modulation of the gut microbiome, and those that addressed important scientific gaps. Eligible articles explored the relationship between obesity and mental health disorders and focused in particular on depression and anxiety. These included pathophysiological explanations involving key factors such as gut microbiota dysbiosis, chronic low-grade inflammation, and intestinal permeability (leaky gut syndrome), as well as outcomes of bariatric surgery. In conducting this narrative review, we considered both original studies (clinical and observational) and narrative reviews or editorials that made relevant theoretical or empirical contributions to expanding the concept of a bidirectional link between the gut microbiome and poor mental health in adult patients. A total of 119 studies met the criteria for this narrative review.

### 2.3. Exclusion Criteria

The narrative review excluded studies involving paediatric populations, exclusively animal models that have no translational relevance to human bidirectional or causal links between obesity, SCFAs and mental health in adult populations, non-peer-reviewed materials such as conference abstracts, posters, or dissertations, and publications that did not address both obesity and mental health components in a clinically meaningful way or that did not conclusively integrate their interaction. We also excluded from our analysis studies that focused exclusively on metabolic or psychiatric outcomes, without examining the bidirectional interaction between obesity and affective symptoms in a detailed manner of circular causality between weight and mental status in adult patients.

### 2.4. Data Selection and Extraction

After eliminating duplicates, titles and abstracts were screened for relevance. Full-length articles were assessed for eligibility based on the inclusion criteria mentioned. We eliminated studies that focused only on obesity and gut microbiota without establishing complex links or if they did not explicitly refer to validated psychiatric disorders such as anxiety, depression, schizophrenia, mood disorders, panic disorder, or bipolar disorder in an integrated context and conditioned by the production of SCFAs and low-grade systemic inflammation. We extracted data on study design, population characteristics, mental health outcomes, obesity-related variables, pathophysiological and causal mechanistic findings.

Currently, the literature on the gut–brain axis and SCFAs is heterogeneous, and given the high variability of available study designs, we opted for a flexible integration of preclinical, clinical, and mechanistic evidence without imposing strict selection criteria. We did not conduct a formal assessment of study quality; instead, we selected peer-reviewed studies for evaluation together, and disagreements were resolved through discussion. Studies with a high impact on the literature were considered as a priority. The studies considered reported on late adolescents and adults (≥16 years), on the bidirectional link between obesity and mental health, and on the mechanisms involved, such as AGCS action, dysbiosis, and inflammation. We prioritized evidence from the literature highlighting the link between the microbiome and interactions with the nervous system or metabolism, and the gaps identified so far in personalized modulation of the gut microbiome, as they contribute to clinical applicability in bariatric surgery decisions and perioperative management. The absence of a pre-registered PROSPERO-type protocol and the high heterogeneity of the analyzed studies constitute methodological limitations of this narrative review, underscoring the need for future standardized longitudinal studies.

## 3. Epidemiological Association Between Obesity and Mental Health

Obesity and mental health disorders, especially depression and anxiety, appear to be associated in a bidirectional manner. Several epidemiological studies have suggested that people who suffer from obesity are more likely to develop neuropsychiatric disorders across the lifespan. This category of people has been reported to show a higher risk of developing anxiety, depression, mood disorders, panic disorder, and bipolar disorder than people with a normal level of body adiposity and a body mass index (BMI) [3,11].

Moreover, the opposite of the previously presented situation might also be viable: patients diagnosed with depression are more likely to adopt a sedentary behaviour, a hypercaloric diet, and a dysfunctional diet based on comfort foods, which can contribute to weight gain and potentially a compromised self-image. Another risk factor for worsening weight status and the appearance of metabolic syndrome may arise from the side effects of antipsychotic medication that are administered to patients with severe depression [11].

In the last two decades, the global impact of obesity has become immense, with over 1 billion people living with obesity in 2024 and an increasing incidence among young people, which has raised many concerns globally and constitutes a public health problem with many challenges. This trend is also reflected in the increasing rate of affective disorders [1]. The comorbidity between obesity and mental health disorders is particularly evident among vulnerable populations, with a predilection for women, people with low socio-economic status, and disadvantaged groups, and patients with chronic metabolic diseases [13,14]. Emotional eating and body image dissatisfaction are important behavioural markers that might predict/anticipate the simultaneous occurrence of anxiety and depression symptoms in overweight and obese individuals [12,15].

Evidence from longitudinal studies suggests that this association is not strictly correlational, but rather reflects shared biological and psychosocial vulnerabilities. Beckman and Harris [16] state that among the risk factors that lead to the amplification and development of mental disorders in obese people are sociocultural factors, stigma, and internalization of negative emotions related to body weight [16].

The study conducted by Skalski-Bednarz et al. [17] demonstrates that emotional eating is a mediating factor in the relationship between depressive symptoms, anxiety, and adipose tissue growth among young women with anorexia nervosa and bulimia. This evidence further strengthens the idea that psychological factors might be taken into consideration when it comes to the connection between obesogenic eating and mental disorders. Similarly, Capoccia et al. [18] identify numerous social and economic factors responsible for unhealthy eating among people with mental disorders and assessed that financial difficulties, social status, stress at work, marginalization, and prolonged exposure to family adversity might be among the associated factors in obesity [17,18].

Even though recent studies identify bariatric surgery as a metabolic solution, the main limitation lies in the psychiatric symptoms, the states of guilt and anxiety that persist even after significant weight loss. This suggests that weight alone does not fully explain psychological distress [4,19]. Thus, the need to consider the link between obesity and mental health as a complex and dynamic association is highlighted, eliminating the concept of “cause-and-effect relationship”.

Body weight and obesity are not linearly correlated with social and economic success, but they can influence each other. Genetic factors, the social environment in early childhood, adopted lifestyle, sedentary behavior, and eating habits learned from the family influence the development of obesity [20]. Regarding the occurrence of depression, it is believed that the impact of genetics on the onset of depression is significantly diminished by a high economic level, that there is an unmediated interaction between genetic vulnerability and poor socio-economic conditions, and that a higher educational level has a protective effect on the occurrence of depression [21]. At the same time, an increased BMI can predispose to depression and can play the role of a predictive factor for mental health, especially in female patients [22]. A significant genetic overlap has been observed between BMI and depression, which confirms the etiological effect of body weight on mental health and the occurrence of depression. These observations have their main origin in analyses of common polygenic architecture and genetic association studies, rather than in direct causal demonstrations. The overlap identified in the literature between loci linked to BMI and susceptibility to depression urgently needs to be interpreted as indicating pleiotropic biological pathways or a possible shared genetic vulnerability, rather than deterministic genetic causality [23]. Effective management of emotional eating in the context of psychological, economic, and emotional stress, and the prevention of this behavior, can be achieved through various means. Cognitive-behavioral therapy, healthy eating, a personalized meal schedule, increased adherence to the Mediterranean diet, and rational food choices contribute to the prevention of depression and overweight, and to the avoidance of food and behavioral patterns that are precursors of these two comorbidities [11].

## 4. Behavioural and Psychological Mechanisms

Several studies have identified behavioural and psychological factors as key contributors to the bidirectional connection between obesity and mental state. One relevant mechanism that might be involved in this interconnection is represented by emotional eating. This is defined as a dysfunctional adaptive response to the environment that predisposes individuals to consume food in response to negative emotions rather than physiological hunger. This behaviour favours both weight gain and the occurrence of higher rates of depressive and anxiety symptoms [11,12,24].

Obese individuals often experience frustration with their body image, which is an important predictor of low self-esteem, internalized shame, and social withdrawal. Thus, these contributors can sustain or perpetuate disorders of the affective spectrum, especially in adolescence and early adulthood [13,15,25]. In terms of stigma based on body weight, both from external sources and self-stigma, it has been shown to amplify emotional disorders and increase the risk of psychiatric complications [26].

The increased incidence of binge eating and depressive symptoms is closely correlated with cognitive distortions, such as dichotomous thinking about food, body shame, and persistent disordered eating behaviours. These behavioural patterns affect body weight management and contribute to the amplification of mood disorders [27,28].

Data from the literature suggest the existence of a feedback loop in which depressive and bipolar disorders are associated with decreased physical activity and sedentary lifestyle, neglect of one’s own image, poor body hygiene, and disturbed circadian rhythm, as well as poor diet quality. These factors favour increased visceral adipose tissue and lead to the development of obesity. On the other hand, obesity might exacerbate mood disorders and promote restlessness, anxiety, and constant worry, fuelling a vicious cycle in which increased weight status and neuropsychiatric disorders amplify each other [29].

Obesity and depression seem to share common physiopathological pathways. This hypothesis led to the idea that the causal approach and clinical context of dysbiosis that generates neuroinflammation should not be viewed in isolation, as proposed by the classical model, but should be analysed in an integrated manner by simultaneously addressing treatment strategies and behavioural or psychological mechanisms that determine both conditions. Having an overview of the two phenomena and the bidirectional relationship, adapted therapeutic strategies could be designed to meet the patient’s needs and to offer a better prognosis for improving metabolic and psychiatric symptomatology [30].

## 5. Biological Mechanisms Involved: Low-Grade Systemic Inflammation and Disruption of Neuroendocrine Signalling Mechanisms

From a causal and pathophysiological point of view, the scientific literature has proposed the existence of numerous factors underlying the alteration of the mental condition in patients who suffer from obesity. Obesity is no longer seen only as an excessive accumulation of adipose tissue with an unattractive appearance and metabolic implications, but also as a source of complex immune responses based on the low-grade inflammation it induces. A diet rich in processed foods and simple sugars might, over time, lead to the intensification of low-grade inflammation associated with the death of adipose cells. This is subsequently responsible for dysregulating neural and humoral signalling mechanisms with effects on both comorbidity factors [31,32].

The current obesity model is characterized by a chronic proinflammatory state, increased levels of circulating cytokines such as interleukin 6 (IL-6), tumoral necrosis factor (TNF-α), or C-reactive protein (CRP), and peripheral insulin resistance leading to metabolic dysfunction and, progressively, the possibility of metabolic syndrome [5,33]. The same causal factors have also been associated with depression, anxiety, and mood disorders, which has strengthened the idea that proinflammatory cytokines in high concentration can alter neurotransmitter metabolism, physiological signalling pathways, and their biochemical synthesis. The scientific literature maintains a particular interest in serotonin, dopamine, and glutamate, which it associates with emotional eating, excessive emotional reactivity, and a state of nervous hyperexcitability [34].

The HPA axis is the body’s main stress response system. People who are diagnosed with either depression or metabolic diseases, such as obesity, may frequently have a dysregulated HPA axis. A multitude of factors can underlie chronic stress and emotional trauma. Thus, chronic stress and emotional suffering might favour a prolonged cortisol secretion, which in turn influences the development of central adiposity. Also, hippocampal neuroplasticity and emotional regulation are affected by increased cortisol levels [9]. In obese patients, depressive symptomatology might be associated with altered levels of kynurenic acid, a metabolite of the kynurenine pathway. Abnormalities and involvement of this pathway are suggested in numerous studies [8,35,36]. These biochemical malfunctions mediate neuroinflammatory cascades and might be responsible for impaired neurogenesis.

Based on the studies analysed, inflammation appears to be both a consequence and a predisposing factor of mood disorders in obesity. Systemic inflammation could be driven by excess adipose tissue in the body, which, in the case of depressed patients, amplifies both their condition and the inflammatory response. This creates a vicious circle that negatively affects both the body’s metabolism and psychological state in adult patients [37,38,39].

**Figure 1:**
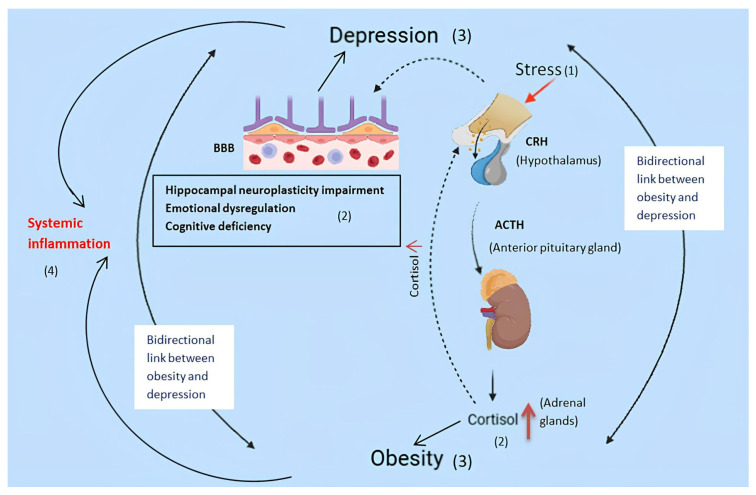
The pathophysiology of the bidirectional link between obesity and depression. Red upward arrows signify the amplification of cortisol levels.

This mutual connection reinforces the hypothesis that obesity and depression are related, being united by common biological and molecular mechanisms, as can be observed in Figure 1. Thus, the need for an integrated therapeutic approach tailored to each patient type is highlighted.

## 6. Gut Microbiota and Intestinal Permeability

Evidence published in recent years attests to the fact that the gut–brain axis has gained considerable attention as a key link between metabolic health and psychiatric well-being (Table 1).

Individuals who experience obesity and associated affective disorders such as depression or anxiety frequently exhibit gut dysbiosis. This is characterized by a marked reduction in microbial diversity and an alteration in the ratio of beneficial to pro-inflammatory species [2,42].

The intestinal microbiota can ferment dietary fibres and produce metabolites that are particularly important in host signalling mechanisms [46]. Considered the main energy substrate for colon cells, SCFAs are synthesized in the intestine and exert important local effects by reducing local and systemic inflammation. The main SCFAs are represented by acetate, butyrate, and propionate. Other SCFAs, such as valerate, isovalerate, lactate isomers, and isobutyrate, are produced in significantly lower quantities. There is a predominance of acetate production among SCFAs (approximately 60% of total SCFAs) [47]. The effects of these compounds are translated at the local level through the beneficial contribution to maintaining intestinal barrier function and preventing local inflammation. Current research addresses the effects of SCFAs at the systemic level. For example, SCFAs might influence the energy consumption of nerve cells, inhibit histone deacetylase (HDAC), and affect the expression of neurotrophic factors, with notable impacts on neuropsychiatric pathology [40,45,48]. It is believed that bacteria that colonize the gastrointestinal tract can modify the synthesis and degradation of neurotransmitters and are involved in communication with the central nervous system (CNS) through complex signalling pathways [49]. SCFAs also play an important role in the functioning of the gut–brain axis. Due to CNS penetration, active and passive mechanisms may be able to modify neuronal behaviour, mitochondrial function, gene expression, and the production of various neurotransmitters [8,44,50].

Most commonly, the decrease in SCFAs production is closely correlated with an inadequate intake of dietary fibre or with the decrease in SCFAs-producing bacterial species, characterized by dysbiosis [51]. In both cases, a decrease in SCFA production is observed, which impacts the intestinal epithelial barrier by compromising its morpho-functional integrity and leading to a condition commonly referred to as ‘leaky gut’. In this context, bacterial lipopolysaccharides (LPS) and other antigens enter the bloodstream, reach the systemic circulation, and may trigger distant inflammatory responses. Dysbiosis associated with prolonged administration of broad-spectrum antibiotics has been recurrently associated with neuroinflammation and disorders such as anxiety or depression due to disruption of the gut–brain axis, affecting neurotransmitter levels and synaptic plasticity [52].

Tryptophan is an essential amino acid that is part of the structure of hormones such as melatonin or serotonin. Its metabolism may be dependent on the health of the intestinal microbiome and may be considered an important causal factor in the pathogenesis of neuropsychiatric diseases, in neurotransmission, and gut–brain communication [53]. Dysbiosis, together with the luminal tryptophan metabolism, reduces its availability for transformation and may, in turn, lead to the appearance of clinical symptoms in neuropsychiatric diseases [54]. Intensely studied in recent years, the link between tryptophan and the occurrence of depression is mainly based on dysregulation of the kynurenine pathway observed in mammals with a metabolism of free circulating tryptophan in plasma [43,55,56].

In addition, gamma-aminobutyric acid (GABA) is produced by bacterial species such as *Lactobacillus rhamnosus*, *Lactobacillus brevis*, *Lactobacillus plantarum*, *Lactobacillus buchneri*, and *Lactobacillus delbrueckii* subsp. *bulgaricus*, *Lactobacillus fermentum*, *Lactobacillus helveticus*, *Lactobacillus paracasei*. Also, species from the genera *Enterococcus*, *Leuconostoc*, *Pediococcus*, *Propionibacterium*, and *Weissella* are considered and associated with major depressive disorders [57,58]. It is believed that altered concentrations and activity of Glutamate (Glu) and GABA are involved in the dysfunction of neurotransmitter signalling mechanisms, which may have a causal link to depression [59]. The GABAergic hypothesis postulates that defects in GABAergic neural inhibition contribute causally to the common phenotypes of depressive disorders, while the glutamatergic hypothesis of depression suggests an association between depressive disorder and elevated glutamate levels [60].

Based on the evidence in the literature, we believe that dysbiosis should not be viewed in isolation as a cause or a consequence of poor mental health but should be viewed in both directions. The gut microbiome is dependent on diet and lifestyle. Seasonal impact and circadian rhythm may be associated with depression [62]. An obesogenic diet rich in sugars and saturated fats contributes to reduced bacterial diversity and dysbiosis, but also to the onset of depression. Stress triggered by the disruption of serotonergic mechanisms through dysbiosis maintains mental disorders. Therefore, dysbiosis is both a cause and a consequence of poor mental health. The diversity of the gut microbiome appears to be inversely proportional to the severity of depressive symptomatology, as an inverse correlation has been established between dietary intake of live microbes and depressive symptoms [63,64]. Obesity and dysbiosis can occur in the context of depression, but they can remodel and amplify depressive symptomatology, the influence being not linear and easy to establish in clinical practice.

Several types of interventions have been proposed in recent years to improve dysbiosis and contribute to the improvement of depressive and anxiety symptoms in patients with SCFAs production deficiency [61,65]. Metabolic interventions such as an increased intake of fish and omega-3, an elevated intake of macro and micronutrients through prebiotics, synbiotics, paraprobiotics, postbiotics, fecal microbiota transplantation and 5-hydroxytryptophan (5-HTP) regulation seem to bring benefits in the improvement of mild to moderate depressive symptoms in the studied populations and to offer therapeutic benefits that are worth considering, but detailed studies are needed to confirm these aspects [66,67,68,69,70].

In the randomized controlled trial conducted by Łoniewski I. et al. [41], it was noted that there is a correlation between the dose of probiotics containing several beneficial bacterial strains and the amount of SCFAs produced. Preliminary, the administration of a higher amount of probiotics leads to a higher level of SCFAs measured in the stool, but future research is needed to confirm this correlation and, eventually, to be able to measure the concentration of SCFAs at the blood level through multiomic analyses, not just by highlighting their concentration in feces [41]. The association of probiotics, prebiotics, fasting, and caloric restrictions in patients with depression seems to offer therapeutic solutions that need to be discussed and developed in the future [71]. Other studies consider that the administration of probiotics in patients with obesity and comorbid depression does not improve depressive symptoms [72]. These mixed points of view offer a heterogeneous vision regarding the efficacy of probiotics among patients with depression, which requires the development of rigorous research protocols, focused on treatment personalization and systemic measurement of these types of metabolites.

In addition, we should note that, in most human studies, SCFA concentrations are measured in fecal samples. This method more specifically reflects microbial fermentation activity in the intestinal lumen than systemic exposure. Fecal SCFA levels do not necessarily correlate with the fractions of SCFA that can cross the blood–brain barrier or with circulating plasma concentrations. However, they may influence neurobiological processes in the brain parenchyma. Interpretations linking SCFA levels to neuropsychiatric effects should be made with caution, taking into account the aforementioned aspects [41]. Future studies will play a key role in the more detailed characterization of systemic SCFA dynamics by integrating plasma metabolomics with multi-omics approaches.

## 7. Bariatric Surgery and Post-Intervention Mental Health

In the case of patients suffering from severe obesity, metabolic and bariatric surgery (MBS) has proven to be one of the most effective methods associated with a marked weight loss and general improvement in health. This provides the premise for its use in the management of neuropsychiatric disorders such as depression and anxiety [13,19]. There is also evidence from the scientific literature of recent years that postulates the worsening of depressive symptoms, suicidal and self-harm tendencies, or drug use disorder following this intervention, which requires careful monitoring of patients and a rigorous selection of cases together with solid evidence and extensive population cohorts that would contraindicate bariatric surgery in this category of people [68,73,74].

MBS proves to be a solution with an impact on the metabolomic profiles of severely obese patients, offering clinical benefits such as remission of type 2 diabetes, weight loss, and improved lipid profile [75]. This combination of advantages, with extended effects on important comorbidities, confirms its impact on cardiovascular, hormonal, and metabolic levels by reducing inflammation and mixed results improving postoperative self-image [40,76]. Some studies report a reduction in depressive symptoms, whereas others indicate no significant differences in emotional state or self-image perception [28]. In contrast, other findings describe markedly negative outcomes, including feelings of self-victimization, heightened anxiety and depressive symptoms, and the experience of multiple forms of stigma (perceived, experiential, anticipated, and internalized) [77].

Some patients continue to experience psychiatric symptoms despite weight loss, and others may even experience emotional instability or suicidal tendencies, especially if pre-existing disorders are not treated before surgery, which may emphasize the need for a preoperative psychological assessment in this category of patients in order to improve the long-term beneficial effects of bariatric surgery. Long-term psychological monitoring and the development of tools adapted to each behavioural pattern are necessary to avoid weight gain and prevent the intensification of anxiety and depression in these patients. Also, in the future, behavioural and psychological factors that may influence the adaptation and return to a normal rhythm of life after bariatric surgery for weight loss, as well as extensive collaboration between nutritionists and psychiatrists or doctors of other specialties, should be taken into account [24,61].

MBS involves complex changes in microbiological and neurohumoral pathways, reflected in a redistribution of the intestinal microbiota postoperatively in patients who have undergone bariatric surgery, but this effect depends on the type of procedure performed [18,78]. In murine models, it has been demonstrated that exposure of experimental animals to bariatric surgery can improve postoperative glycaemic control, but in humans, non-invasive methods are needed that do not alter intestinal permeability or contribute to dramatic reductions in the number of SCFAs-producing species [79,80].

Postoperatively, patients who have undergone bariatric surgery exhibit marked weight loss and significant metabolic improvements. Inflammatory markers decrease following surgery, and the level of low-grade inflammation decreases significantly, providing a basis for lower production of proinflammatory cytokines and allowing for reduced neuroinflammation and anxiety, depression, and mood disorders [74,80]. However, MBS requires careful, personalized use for each patient type, as its disadvantages are considerable, including malabsorption, endocrine dysregulation, hypoglycaemia, and nesidioblastosis, and must be taken into account when opting for this type of surgery [19,36]. Perioperative monitoring over long periods is a first-line necessity in the management of this type of patient and requires analysis of the risk-benefit ratio [3].

Evidence regarding increased suicide rates, mental disorders, and especially depression among patients who have undergone bariatric surgery is inconsistent and heterogeneous, requiring confirmation by large cohort studies. In fact, the evidence to date is not definitive regarding a linear link between bariatric surgery and increased suicide attempts. The risk of suicide may be higher postoperatively compared to the non-surgical cohort. The risk of suicide attempt is 1.64 times higher after bariatric surgery. Although the absolute incidence remains relatively low in both groups (2.2% versus 1.3%), the 64% excess risk highlights the need for specific psychiatric monitoring after surgery, indicating significant postoperative vulnerability. This aspect is also due to the behavior adopted by the patient, namely the medication they are taking or the addictions they are facing [81]. The recently published study by Roger S. McIntyre [82] reiterates the idea that there are reports of cases undergoing bariatric surgery with an increased risk of developing alcohol abuse and with the appearance of depression or suicidal tendencies [82]. Self-harm and suicidal thoughts may be present preoperatively, which increases the need for thorough screening and careful and responsible multidisciplinary management of the medical case. It is believed that self-harm has not increased following bariatric surgery, and to the extent that cases have been reported, they must be correlated with the additional psychopathology that the patient was facing prior to the intervention [83]. Contrary to this hypothesis, another study considers that self-harm increased up to fourfold among patients who underwent such an intervention and that it is correlated with the social context, younger age, and psychiatric comorbidities. The risk of self-harm is approximately four times higher in patients undergoing bariatric surgery than in the general population [84]. The occurrence of mental disorders and the recovery of well-being is correlated with the level of self-compassion. Thus, psychological and emotional recovery is dependent on the level of self-compassion.

In patients who have adopted a self-compassionate attitude, better emotional compensation is observed than in those with a lower level of self-compassion [85]. Another aspect discussed is the tendency to take refuge in various forms of addictions, such as alcohol or psychotropic substances. A modest but constant increase in the prevalence of abuse of various substances and alcohol is observed in patients who have undergone bariatric surgery, a higher tendency than in patients who have opted for GLP-1RA therapy [86]. The data are not uniform, and large, dedicated randomized clinical trials are needed to confirm these aspects accurately before any therapeutic recommendation.

The attempt to evaluate the change in problematic alcohol and substance use after bariatric surgery based on a survey could not accurately indicate a notable increase in this tendency [87]. A correlational analysis is required in the future to attest to the validity of these data and to overcome the non-specific and subjective nature of the questionnaire method by integrating more objective indicators or real-time evaluation methods. A real future direction is the attempt to establish a concrete link between the mood after surgery and the tendency to consume, respectively, abuse alcohol and food [88]. Even though bariatric surgery currently remains one of the most effective forms of treatment for weight loss, the long-term socio-emotional and psychological consequences must be taken into account, explained to the patient and their relatives, and postoperative psychotherapeutic intervention must be promoted through mixed teams to ensure that the patient’s perception of the new appearance, their new social image, and their encouragement to seek specialized support [89].

## 8. The Bidirectional Link Between Obesity and Mental Disorders

Several studies have confirmed that obese individuals are up to 70% more likely to develop psychiatric comorbidities compared to normal-weight individuals [90,91]. Depression is the most frequently studied psychiatric consequence in the context of obesity. The most common symptoms include persistent sadness, lack of energy, sleep disturbances, and cognitive impairment. The perception of one’s own body weight plays a crucial role in the development of disordered eating habits and in accentuating the emotional disorder installed in these patients. Adolescents and women appear to be particularly vulnerable, with significantly higher rates of emotional eating and mood disorders in response to weight-related stigma [12,92].

Both depression and obesity are associated with altered signalling through glucocorticoid hormones. The increased levels of circulating proinflammatory cytokines may affect blood–brain barrier (BBB) integrity, induce chronic inflammation of the brain parenchyma, and alter interneuronal communication by reducing synaptic plasticity and diminishing neurotransmitter signalling in the CNS [2,93]. The persistence of proinflammatory cytokines in the circulation stimulates glial cells, especially microglia. There is also a dysregulation of signalling through hunger and satiety hormones (ghrelin and leptin), which explains the patient’s compulsive hunger, but is not justified by a real physiological need that must be satisfied [5,38].

Recent medical neuroimaging techniques have allowed the evidence of structural and functional pathological changes in the brain, thus strengthening the dual connection between obesity and emotional disorders. In a recent research, Zhang et al. [94] analyzes a series of 24 studies focusing on neuroimaging and brain-level changes. Thus, these studies have revealed significant reductions in grey matter density (especially in the frontal and temporal regions and in the thalamic and hypothalamic regions) and white matter structure in patients with comorbid obesity and depression. Regarding brain functionality, changes in neuronal activity can be observed in regions that are involved in emotional regulation, cognitive control, and the reward process. The observed changes strongly suggest that the link between obesity and depression is tied together through pathological neuroplasticity processes that could accentuate psychological stress and eating disorders [78,94]. Excessive adiposity alters CNS function through the synergistic pathophysiological action of systemic low-grade inflammation, BBB permeability, and the progressive development of insulin resistance. Cumulatively, these can disrupt the neural circuits involved in mood regulation and lead to neuropsychiatric tension manifested by agitation, anxiety, and prolonged restlessness. These pathophysiological changes may explain the high comorbidity between obesity and neuropsychiatric disorders, justifying the occurrence of cognitive decline to the extent that the symptomatology does not remit in this type of person. In addition, obesity-related hormonal imbalances and gut-derived neuroactive metabolites could considerably influence neurodevelopmental trajectories and emotional processing [40,95].

## 9. The Role of Intestinal Dysbiosis and SCFAs in Brain Function

In maintaining neuronal and emotional homeostasis, the intestinal microbiota plays a key role. The intestinal microbiota maintains this homeostasis through the SCFAs it produces. Butyrate plays the most important role. These metabolites play a role in neuroprotection and have anti-inflammatory and metabolic regulatory roles. SCFAs cross the BBB and interact with glial cells. They are also involved in modulating gene expression through mechanisms involving G protein-coupled receptors such as GPR41 and GPR43, as well as the inhibition of HDAC. It is believed that butyrate has properties that enhance the morphological integrity of the BBB and reduce neuroinflammation. This mechanism is achieved by increasing the expression of tight junction proteins, such as claudin-5 and ZO-1 [38,96,97]. Studies show that, particularly in obese people, intestinal dysbiosis is present, leading to decreased production of SCFAs, compromised intestinal barrier integrity, and reduced functionality. The presence of systemic inflammatory processes impairs cognitive and emotional processes [2,10]. A diet low in fibre and high in fat may favour the development of intestinal dysbiosis, which would manifest as increased intestinal permeability and systemic inflammation. Also, the translocation of LPS and their crossing of the BBB, where they would interact with glial cells, might promote the development of a neuroinflammatory response [98,99,100].

Changes in gut flora are associated with reduced levels of beneficial SCFA-producing bacteria, leading to lower SCFA levels and increased pro-inflammatory markers. *Faecalibacterium* and *Roseburia* are two SCFA-producing bacteria whose alterations have been documented in people with psychiatric pathologies such as depression and schizophrenia [76,101,102].

The roles of SCFAs on the CNS are achieved both directly and indirectly. They cross the BBB directly and interact with receptors on neuroglia. Indirectly, SCFAs influence gut hormones and vagal signalling. Butyrate may modulate gene expression by inhibiting HDACs and promoting the expression of tight junction proteins that maintain BBB integrity [103,104]. The cumulative effect of these mechanisms is to reduce brain inflammation by contributing to emotional resilience. SCFA might also modulate the HPA axis, and by influencing glucocorticoid feedback, SCFA could reduce hypercortisolaemia. The effects of this mechanism consist of preventing hippocampal lesions and constitute a relevant and particularly useful mechanism for patients with obesity and mood disorders [38,74,105]. Butyrate might affect dopamine receptor signalling, which also makes it involved in reward processing and emotional regulation [75,106].

## 10. Chronic Inflammation, Leaky Gut Syndrome, and Brain Vulnerability

Chronic inflammation, together with alterations involving glucocorticoid signalling pathways, is the main pillar of the link between obesity and depression. The integrity of the BBB is affected by its interaction with increased levels of TNF-α, IL-6, and CRP. This results in a neuroinflammatory state that involves synapses and affects neuronal communication [2,33]. This inflammatory cascade activates microglial cells and disrupts the balance of appetite-regulating hormones, such as leptin and ghrelin, perpetuating compulsive eating and mood instability [5,38].

Research shows that high-fat diets, often associated with obesity, might be responsible for intestinal dysbiosis and contribute to increased intestinal permeability and systemic endotoxemia [10,107]. Intestinal dysbiosis worsens this process by weakening the intestinal epithelial barrier and leading to “leaky gut syndrome,” which contributes to increased levels of circulating endotoxins, such as LPS. These endotoxins trigger immune activation and stimulate the release of inflammatory mediators, ultimately impairing synaptic plasticity and disrupting neurotransmitter [42,98]. In addition, the depletion of SCFAs resulting from the loss of key bacterial taxa (e.g., *Faecalibacterium*, *Roseburia*) also impairs anti-inflammatory signalling, thereby significantly reducing the brain’s resistance to inflammatory insults [95,108].

**Figure 2:**
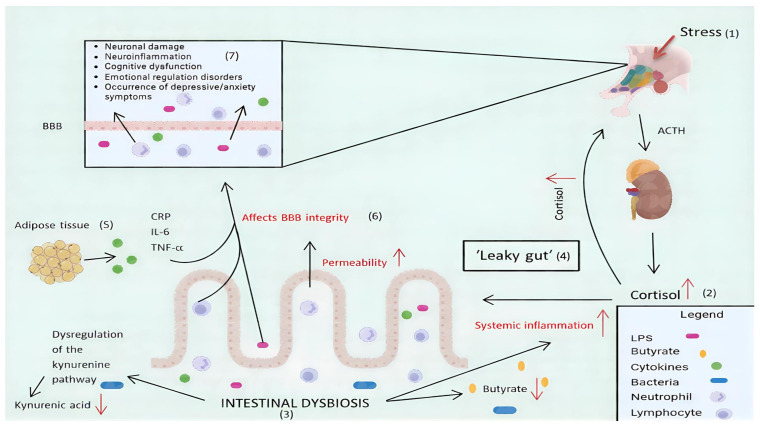
The implications of intestinal dysbiosis in the occurrence of mental illnesses. Red downward arrows indicate a decrease or reduction, while red upward arrows signify an increase.

Recent studies indicate that dysregulated signalling between the gut and the brain can further disrupt hippocampal plasticity, emotional regulation, and HPA axis feedback, thus worsening both mood disorders and the person’s cognitive abilities [104,109]. Microbiota-derived metabolites not only modulate immune responses but may also engage in complex interactions with microglia and astrocytes, and amplify neuroinflammation in the setting of dysbiosis, as illustrated in Figure 2.

In addition, impaired microbial metabolism may influence the kynurenine pathway. This aspect has emerged as a relevant link between chronic inflammation and neuropsychiatric outcomes in patients. Intestinal dysbiosis interacts with tryptophan metabolism. Tryptophan metabolism could be deregulated and directed, in this way, towards the production of toxic metabolites of kynurenine, which lead to the appearance of depressive symptoms and cognitive deficiencies [34,35]. The combined impact of increased intestinal permeability, immune activation, and disrupted metabolism of SCFAs and tryptophan contributes to the onset and progression of depression and anxiety, and to significant cognitive dysfunction, especially in obese individuals [2,5,95].

## 11. Hedonic Eating and Dopaminergic Dysregulation

Hedonic eating is presented as the process of consuming foods rich in fat and sugar, with the primary aim of obtaining pleasure and satisfaction, without necessarily involving physiological hunger. This process is prevalent in people with obesity and mood disorders. It is connected to an alteration of the mesolimbic dopamine-dependent reward system, incriminated by a possible reduction in the sensitivity of dopamine D2 receptors in the nucleus accumbens [94,110]. Downregulation of the D2 receptor is also associated with compulsive food-seeking behaviours. These disorders are observed particularly in people with tendencies towards binge eating and emotional dysregulation [97,111]. High-fat diets are considered to lead to an alteration of the corticotropin-releasing factor (CRF) signalling pathway, leading to increased dopamine turnover in the lateral septum, which will lead to a dysregulation of the reward process and emotional processes [77]. Emerging neurogenetic models suggest that such maladaptive eating patterns may reflect a broader “reward deficit syndrome” (RDS) in which reduced dopamine tone contributes to chronic hedonic seeking and impaired satiety signalling [112,113]. In addition, butyrate can influence the homeostasis of the dopaminergic system [7,38]. Thus, it modulates the expression of genes encoding components of dopaminergic neurotransmitter-dependent circuits, thereby favouring neuroplasticity. Addictive-like behaviours could be reinforced by dysbiosis and decreased production of SCFAs, through the decline of the homeostatic balance mechanism described above.

Some recent neurobiology studies hypothesize that neurons that expressing cholecystokinin receptors and processing satiety signals interact with the hedonic pathway to regulate meal sizes and food reward. These findings provide valuable additional insights into how gut–brain signalling contributes to overeating [114,115]. In addition, dietary strategies and mindfulness-based interventions have shown promise in modulating reward circuitry and reducing emotional and hedonic eating in clinical settings [116,117]. Emotional distress and social stigma further amplify hedonic eating patterns, creating a vicious cycle between food consumption, mood swings and weight gain [30,118].

## 12. Clinical Implications and Future Research Directions

Our reviewed evidence suggests that clinicians should adopt a more integrative and multidisciplinary approach to treating individuals with both obesity and mental health disorders. After reviewing the relevant research, it is clear that the findings are quite varied and, at times, even conflicting, largely due to the different study designs employed. Specifically, there is a notable lack of consensus on the effectiveness of probiotics, fluctuations in SCFA levels, and psychiatric effects observed following bariatric surgery. These discrepancies likely stem from differences in the populations studied, particular methodological approaches, the predominance of observational or exploratory designs in this field, and the constantly evolving techniques for assessing the microbiome. Although several mechanistic pathways linking SCFA and the gut microbiota to short- and long-term neuropsychiatric conditions have been proposed, these relationships are currently best interpreted as hypothesis-generating rather than definitively validated and objectified. Longitudinal studies and controlled clinical trials are needed to reconcile these discrepancies and clarify causal pathways in a standardized and precise manner. Routine psychological screening is recommended in obese patients, especially before bariatric surgery, because pre-existing mood or anxiety disorders could influence both the evolution, prognosis, and outcome of treatment, impacting the patient organically and socially.

Similarly, nutritional, psychological, and psychiatric interventions should no longer be considered in isolation. They should be considered as interconnected elements of a patient-centred care model in which the particularity of each case and the special requirements of each sick person are not addressed individually and in isolation, but in close bio-psycho-social correlation with the social, environmental, and behavioural factors that led to the worsening of the clinical context the patient is facing.

Gut microbiota profiling and targeted dietary interventions—by increasing fibre intake, using probiotics or prebiotics, and modulating SCFAs-producing bacteria—could complement the traditional pharmacological and psychotherapeutic treatments currently used. Modulation of SCFAs could represent a novel and non-invasive adjunctive strategy in the management of mood disorders in obese individuals, which is why it deserves consideration in the future. Given the overlapping roles of systemic inflammation and dopaminergic dysregulation, a future direction is to increase attention to early identification of neuroinflammatory markers and reward system dysfunction in high-risk populations.

Although the number of publications correlating SCFAs, gut microbiota, and neuropsychiatric outcomes is increasing, recent promising evidence has highlighted important gaps in our understanding of this relationship. Larger longitudinal studies are needed to better establish causality between SCFA depletion, dysbiosis, and mental health deterioration in adult patients with both comorbidities.

Regarding therapeutic interventions targeting the gut flora (probiotics, prebiotics, symbiotics, faecal transplantation), their efficacy should be verified by randomized clinical trials. We also emphasize the need to identify biomarkers characterizing SCFAs levels, intestinal barrier integrity, and neuroinflammation. Integrating these aspects could contribute to early diagnosis and personalized patient care.

Among the areas that need to be developed in the future are studies detailing the importance of, and how, gender, age, diet, and genetics interact with the intestinal microbiota to influence vulnerability to the development of obesity and mental illness. In this way, we highlight the impact of these factors on the life and social integration of those types of patients. Studies that evaluate changes in circuits regulating stress and reward following modulation of the microbial flora or diet could help identify potential mechanisms. Imaging could also be used to observe the effects of treatment and to improve it.

As knowledge about the gut–brain axis multiplies and becomes more concrete, we would move from correlation and causal analysis of these elements to therapeutic interventions and their effectiveness. Understanding the mechanisms by which microbiota metabolites influence brain development, neuroplasticity, and behaviour could pave the way for new and innovative therapeutic strategies for the treatment of obesity and depression.

A multitude of studies have been conducted on obesity and depression, but a limited number have comprehensively integrated psychological factors, pathophysiological mechanisms, and the role of the brain–gut axis. Regarding current information on anxiety, unsatisfactory body image, and mental health after MBS, the data are limited, fragmented, and heterogeneous. We also included numerous studies in our analysis that present a wide diversity in terms of ethnicity and age, suggesting variable results. The aspects described may limit our study’s ability to reach a clear conclusion on the topic.

## 13. Conclusions

The link between obesity and depression is extremely complex, broad, and bidirectional, being achieved through multiple physiological pathways. The main pathway involved is formed by the brain–gut axis and its mediators (SCFAs). These play essential roles in maintaining intestinal integrity, modulating neuroinflammation, and regulating neurotransmitter signalling. Dysbiosis and decreased levels of beneficial SCFAs are implicated in the production of BBB dysfunction, low-grade systemic inflammation, and dopaminergic dysregulation.

All of these abnormal changes contribute to the emergence and exacerbation of mood and cognitive disorders with a marked impact on all levels. The vicious cycle between obesity and poor mental health is exacerbated by emotional eating, social stigma, and hedonic dysregulation. This evidence highlights the importance of adopting an integrative biopsychosocial archetype in both clinical practice and medical research. Future prevention and intervention strategies should target gut microbiota composition, SCFA production, and the neuroinflammatory response, along with personalized behavioural and psychological support for each patient.

Although promising, this field is still in its infancy and requires considerable improvement to be fully understood. More studies are needed to fully understand the mechanism by which gut microbiota-derived metabolites influence brain function and behaviour. A more distinctive and detailed taxonomic characterization of bacterial species associated with the development of psychiatric diseases could lead to the development of personalized therapies for patients suffering from obesity and mental illness.

## Figures and Tables

**Table 1:** Key Evidence Linking Gut Microbiota, Short-Chain Fatty Acids, Intestinal Permeability, and Mental Health in Obesity.

Authors	Study Type	OCEBM Level	Main Objective	Main Findings	Key Methodological Limitations
Silva et al., 2020 [40]	Systematic review & meta-analysis	Level 1	To analyse associations between depression subtypes and obesity	Confirmed association between obesity and specific depressive phenotypes	High heterogeneity; observational primary studies
Łoniewski et al., 2023 (cited in text) [41]	Randomized controlled trial	Level 2	To evaluate probiotic dose–SCFA relationship	Higher probiotic dose correlated with increased fecal SCFAs	SCFAs measured only in stool; limited psychiatric endpoints
Kovtun et al., 2022 [42]	Clinical observational study	Level 4	To characterize gut microbiota composition and neurometabolic profile in MDD	Observed correlation between microbial diversity and altered neurometabolic signatures in MDD	Cross-sectional design; no causal inference
Shin & Cho, 2020 [43]	Cross-sectional observational study	Level 4	To compare microbiota profiles in obese vs. normal-weight children	Preliminary evidence of shifted bacteroidetes ratio in obese children	Pediatric population; no psychiatric evaluation
Zhong et al., 2023 [44]	Animal experimental study	Level 5	To assess neurotransmitters and SCFAs in dysbiosis-induced rats	Experimental dysbiosis appeared to modulate SCFAs and neurotransmitter levels	Animal model; limited human applicability
Fillier et al., 2022 [45]	In vitro experimental study	Level 5	To assess neuronal effects of systemic SCFAs	SCFAs impaired lipid metabolism and induced neuronal apoptosis	In vitro model; uncertain physiological relevance
Cui et al., 2025 [46]	Narrative review	Level 5	To summarize microbiota-mediated mechanisms in fat deposition	Point toward a role of gut microbiota in modulating inflammation and adiposity	No primary data; descriptive synthesis
Fusco et al., 2023 [47]	Narrative review	Level 5	To describe SCFA-producing bacteria and physiological roles	SCFAs appeared to modulate barrier integrity and inflammation	Mechanistic focus; limited clinical outcomes
Zou et al., 2025 [48]	Narrative review	Level 5	To evaluate omega-3 effects on gut microbiota	Omega-3 was associated with microbiota composition and inflammation	Limited psychiatric outcome data
Dicks, 2022 [49]	Narrative review	Level 5	To explore gut bacteria–neurotransmitter interactions	Potential pathways for microbiota-driven synthesis of GABA, serotonin, and dopamine	Predominantly experimental data
Lan et al., 2024 [50]; Zhao et al., 2025 [51]	Bibliometric & narrative reviews	Level 5	To map SCFA research in CNS diseases and systemic homeostasis	Expanding research linking SCFAs to neuroinflammation	No outcome-based clinical trials
Cortés et al., 2025 [52]	Narrative review	Level 5	To analyse microbiome–immune interactions	Dysbiosis may contribute to systemic and neuroinflammation	Broad scope; limited disease specificity
Hou et al., 2023 [53]; Gao et al., 2020 [54]	Narrative reviews	Level 5	To explore tryptophan metabolism in gut–brain communication	Dysbiosis was linked to altered tryptophan availability and kynurenine pathway	Mechanistic framework
Mándi et al., 2022 [55]; Brown et al., 2021 [56]	Review & editorial	Level 5	To assess kynurenine pathway in depression	Kynurenine imbalance associated with depressive symptoms	Limited interventional evidence
Fashogbon et al., 2024 [57]	Narrative review	Level 5	To review microbial GABA production	Identification of GABA-producing bacterial strains	Functional psychiatric outcomes not assessed
Tette et al., 2022 [58]; Liwinski et al., 2023 [59]	Experimental & narrative studies	Level 5	To evaluate microbial GABA in stress and depression	Lactobacillus-derived GABA could modulate the HPA axis	Limited large-scale clinical trials
Cutler et al., 2023 [60]; Dyndał et al., 2025 [61]	Narrative reviews	Level 5	To explore metabolic and GABAergic modulators in depression	Metabolic and neurotransmitter pathways interconnected	Predominantly pharmacological focus

Levels of evidence classified according to the Oxford Centre for Evidence-Based Medicine (OCEBM) 2011 hierarchy.

## Data Availability

No new data were created or analysed in this study. Data sharing is not applicable to this article.

## Abbreviations

The following abbreviations are used in this manuscript:

5-HTP	5-hydroxytryptophan
BBB	Blood–brain barrier
BMI	Body mass index
CNS	Central nervous system
CRF	Corticotropin-releasing factor
CRP	C-reactive protein
GABA	Gamma-aminobutyric acid
Glu	Glutamate
HDAC	Histone diacetylase
HPA	Hypothalamic–pituitary–adrenal
IL-6	Interleukin 6
LPS	Bacterial lipopolysaccharides
MBS	Metabolic bariatric surgery
RDS	Reward deficit syndrome
SCFAs	Short-chain fatty acids
TNF-α	Tumoral necrosis factor
